# Supplementary material for: Detailed molecular and epigenetic characterization of the pig IPEC-J2 and chicken SL-29 cell lines
Source: iScience. 2023 Feb 20;26(3):106252. doi: 10.1016/j.isci.2023.106252 (PMC10018572; doi:10.1016/j.isci.2023.106252)
Supplement: Data S2. Complete homer output for identified motifs in Chicken SL-29, related to Tables 5 and 6 — Homer motif analysis results for histone modifications H3K4me1, H3K4me3, H3K27ac, enhancers, and ATAC-seq of chicken SL-29 cell line. Parameters for possible false positives is as mentioned earlier for S5. [file mmc3.zip › Data_S2/S6/Chicken_SL_29/peak_files_enhancers/homerResults.html]

peak\_files\_enhancers/ - Homer de novo Motif Results


# Homer *de novo* Motif Results (peak\_files\_enhancers/)

Known Motif Enrichment Results  
Gene Ontology Enrichment Results  
If Homer is having trouble matching a motif to a known motif, try copy/pasting the matrix file into
STAMP  
More information on motif finding results: HOMER
| Description of Results
| Tips
  
Total target sequences = 18518  
Total background sequences = 31552  
\* - possible false positive  

|  |  |  |  |  |  |  |  |  |
| --- | --- | --- | --- | --- | --- | --- | --- | --- |
| Rank | Motif | P-value | log P-pvalue | % of Targets | % of Background | STD(Bg STD) | Best Match/Details | Motif File |
| 1 | A G C T A C T G C G T A A T C G A C G T G T A C C G T A A G C T | 1e-525 | -1.210e+03 | 17.19% | 6.59% | 80.4bp (86.5bp) | Smad2::Smad3/MA1622.1/Jaspar(0.977) More Information \| Similar Motifs Found | motif file (matrix) |
| 2 | T C G A T C A G A C T G T C G A C G T A T C G A G A C T C A T G T G C A A G T C | 1e-91 | -2.104e+02 | 12.92% | 8.47% | 82.7bp (85.9bp) | EWS:ERG-fusion(ETS)/CADO\_ES1-EWS:ERG-ChIP-Seq(SRA014231)/Homer(0.862) More Information \| Similar Motifs Found | motif file (matrix) |
| 3 | T C G A T C G A G T A C G T C A C G A T T A G C C G A T A C T G A C T G C A G T | 1e-42 | -9.801e+01 | 17.07% | 13.50% | 85.1bp (87.5bp) | TWIST1/MA1123.2/Jaspar(0.897) More Information \| Similar Motifs Found | motif file (matrix) |
| 4 | C G A T A C T G A C T G G C T A C G T A A C G T A C T G A G C T | 1e-41 | -9.444e+01 | 16.82% | 13.33% | 83.5bp (86.9bp) | TEAD3/MA0808.1/Jaspar(0.980) More Information \| Similar Motifs Found | motif file (matrix) |
| 5 | G T C A A C G T A C T G C G T A A C G T A C T G A G T C C G T A C G T A A G C T | 1e-30 | -7.030e+01 | 1.32% | 0.57% | 82.7bp (88.1bp) | ATF4/MA0833.2/Jaspar(0.983) More Information \| Similar Motifs Found | motif file (matrix) |
| 6 | A C T G A G T C C G A T A C G T C T A G C A T G C T A G C G T A C T A G A C G T C G T A C G T A A C T G | 1e-27 | -6.443e+01 | 0.12% | 0.01% | 77.1bp (30.7bp) | PRDM1/MA0508.3/Jaspar(0.603) More Information \| Similar Motifs Found | motif file (matrix) |
| 7 | G C T A C G T A A G T C G T A C C T A G A T G C G T C A C G A T | 1e-27 | -6.275e+01 | 24.34% | 21.01% | 85.6bp (87.1bp) | RUNX2/MA0511.2/Jaspar(0.871) More Information \| Similar Motifs Found | motif file (matrix) |
| 8 | A T C G C G A T T G A C A T G C A G T C T C G A C T A G T G A C | 1e-24 | -5.746e+01 | 26.67% | 23.38% | 84.2bp (90.0bp) | ZNF416(Zf)/HEK293-ZNF416.GFP-ChIP-Seq(GSE58341)/Homer(0.824) More Information \| Similar Motifs Found | motif file (matrix) |
| 9 | C G T A A G T C A C T G A C T G A C T G A C T G A C T G A C T G C G T A A C T G A G T C A C G T A G T C | 1e-24 | -5.713e+01 | 0.11% | 0.01% | 78.7bp (61.2bp) | PB0204.1\_Zfp740\_2/Jaspar(0.705) More Information \| Similar Motifs Found | motif file (matrix) |
| 10 | G A T C G A T C G C A T C A G T A C G T A T C G A C G T A G T C C G A T A C T G G A T C C T A G A T C G | 1e-24 | -5.713e+01 | 0.11% | 0.00% | 77.8bp (0.0bp) | PB0166.1\_Sox12\_2/Jaspar(0.790) More Information \| Similar Motifs Found | motif file (matrix) |
| 11 | T G A C T A G C A T C G A C T G G C T A T G C A A T C G A G C T T A C G T A C G | 1e-23 | -5.314e+01 | 3.18% | 2.06% | 83.6bp (91.9bp) | ZBTB7A/MA0750.2/Jaspar(0.856) More Information \| Similar Motifs Found | motif file (matrix) |
| 12 | C A T G A G T C G A T C G A C T A G T C A G T C G T C A T A G C T G C A A T G C G T A C C T A G A T C G | 1e-20 | -4.626e+01 | 0.15% | 0.02% | 75.8bp (101.7bp) | ZNF354C/MA0130.1/Jaspar(0.634) More Information \| Similar Motifs Found | motif file (matrix) |
| 13 | T G A C G C T A A C T G G T A C T A C G T G C A A G T C A C G T A T G C G A T C G T A C G C T A A T G C | 1e-19 | -4.378e+01 | 0.15% | 0.01% | 78.8bp (53.7bp) | ZNF75D/MA1601.1/Jaspar(0.720) More Information \| Similar Motifs Found | motif file (matrix) |
| 14 | C G T A A C G T C G T A A G T C A G T C A G T C C G T A A C G T A C G T A G T C A G T C A G T C A C G T | 1e-18 | -4.312e+01 | 0.09% | 0.01% | 76.0bp (31.6bp) | TEAD2/MA1121.1/Jaspar(0.595) More Information \| Similar Motifs Found | motif file (matrix) |
| 15 | A C G T A C T G A G T C C A G T A C T G A G C T A T C G C A G T A G T C C T G A A G C T G A C T A T C G | 1e-18 | -4.290e+01 | 0.13% | 0.01% | 90.0bp (95.6bp) | Nfe2l2/MA0150.2/Jaspar(0.805) More Information \| Similar Motifs Found | motif file (matrix) |
| 16 | G T C A C A G T C A T G G T C A C G T A C G A T C A G T G A C T C T G A C A G T A C T G T G C A A G T C | 1e-18 | -4.290e+01 | 0.13% | 0.01% | 91.4bp (24.2bp) | PB0171.1\_Sox18\_2/Jaspar(0.703) More Information \| Similar Motifs Found | motif file (matrix) |
| 17 | A C T G A G T C A C T G A G T C A C T G A C T G G T A C A G T C A G T C C G T A | 1e-18 | -4.202e+01 | 0.22% | 0.04% | 76.7bp (107.4bp) | KLF3/MA1516.1/Jaspar(0.670) More Information \| Similar Motifs Found | motif file (matrix) |
| 18 | T A G C G C A T T G C A T G C A T A G C T A C G T A C G T G A C T G A C A T C G | 1e-17 | -4.133e+01 | 8.04% | 6.41% | 83.1bp (89.8bp) | MYB(HTH)/ERMYB-Myb-ChIPSeq(GSE22095)/Homer(0.803) More Information \| Similar Motifs Found | motif file (matrix) |
| 19 | A C G T A G T C C G T A A G T C C G T A A C G T A C T G A G T C C T G A C G T A | 1e-16 | -3.831e+01 | 0.54% | 0.20% | 86.1bp (92.3bp) | MXI1/MA1108.2/Jaspar(0.765) More Information \| Similar Motifs Found | motif file (matrix) |
| 20 | G A T C C G A T C T A G A T G C G T A C G C T A T C G A C T G A T A G C A C G T C G T A C T A G G T C A | 1e-15 | -3.644e+01 | 0.08% | 0.01% | 60.7bp (42.0bp) | NF1:FOXA1(CTF,Forkhead)/LNCAP-FOXA1-ChIP-Seq(GSE27824)/Homer(0.676) More Information \| Similar Motifs Found | motif file (matrix) |
| 21 | A G T C C G A T A T G C G C A T A T C G A G T C A T C G T A G C G T C A A C G T A T C G A T G C C T A G | 1e-15 | -3.525e+01 | 0.18% | 0.03% | 81.3bp (66.3bp) | NRF1(NRF)/MCF7-NRF1-ChIP-Seq(Unpublished)/Homer(0.814) More Information \| Similar Motifs Found | motif file (matrix) |
| 22 | T A G C C G T A A G T C C G T A A G T C G A T C T A C G G T C A G T A C A C G T A C G T A T G C C G T A | 1e-15 | -3.518e+01 | 0.11% | 0.01% | 98.9bp (38.2bp) | KLF9/MA1107.2/Jaspar(0.685) More Information \| Similar Motifs Found | motif file (matrix) |
| 23 | A C G T C G A T A G C T A C G T G A C T A T G C G T A C G T A C | 1e-15 | -3.480e+01 | 21.25% | 18.92% | 85.3bp (87.8bp) | NFATC2/MA0152.1/Jaspar(0.791) More Information \| Similar Motifs Found | motif file (matrix) |
| 24 | A C T G A C G T C T G A A C T G A C G T A C G T C G T A A C T G A C G T A C G T | 1e-15 | -3.469e+01 | 0.17% | 0.03% | 72.8bp (66.8bp) | HOXD3/MA0912.2/Jaspar(0.689) More Information \| Similar Motifs Found | motif file (matrix) |
| 25 | A C T G C G T A A C T G A C T G A G T C A C T G A C T G A C T G C G T A C G T A | 1e-15 | -3.462e+01 | 0.10% | 0.01% | 71.0bp (71.7bp) | E2F6/MA0471.2/Jaspar(0.900) More Information \| Similar Motifs Found | motif file (matrix) |
| 26 | A T G C C T A G A T G C A T G C A T C G T A G C A T G C T C G A G A C T A T C G A C T G A G T C A T C G | 1e-13 | -3.152e+01 | 1.07% | 0.60% | 82.4bp (85.3bp) | YY2/MA0748.2/Jaspar(0.829) More Information \| Similar Motifs Found | motif file (matrix) |
| 27 | T G C A T G C A C G A T A C T G G T A C C G A T A C T G G A T C G T A C G T C A G A T C C T A G A G C T | 1e-13 | -3.026e+01 | 0.10% | 0.01% | 66.3bp (73.7bp) | MAFF/MA0495.3/Jaspar(0.674) More Information \| Similar Motifs Found | motif file (matrix) |
| 28 | A C G T G T A C A C G T A C T G C G T A C G T A A C T G A G T C A C T G C G T A | 1e-12 | -2.921e+01 | 0.09% | 0.01% | 97.1bp (38.0bp) | POL008.1\_DCE\_S\_I/Jaspar(0.651) More Information \| Similar Motifs Found | motif file (matrix) |
| 29 \* | A C G T C G T A C G T A A G T C A C G T A C G T A C T G A C G T C G T A A G T C | 1e-10 | -2.404e+01 | 0.08% | 0.01% | 76.0bp (16.6bp) | PH0085.1\_Irx4/Jaspar(0.676) More Information \| Similar Motifs Found | motif file (matrix) |
| 30 \* | A C T G A G T C C G T A C G T A A G T C C G T A A G T C A G T C A C T G A G T C | 1e-10 | -2.404e+01 | 0.08% | 0.01% | 88.2bp (73.2bp) | PB0117.1\_Eomes\_2/Jaspar(0.708) More Information \| Similar Motifs Found | motif file (matrix) |
| 31 \* | A C T G A G T C A C T G C G T A C G T A A C G T C G A T A C T G C T A G A C T G | 1e-10 | -2.327e+01 | 0.09% | 0.01% | 78.6bp (60.1bp) | PH0089.1\_Isx/Jaspar(0.689) More Information \| Similar Motifs Found | motif file (matrix) |
| 32 \* | A C G T A C G T C G T A C G T A A C T G G T C A A C T G A C G T A C G T A G T C | 1e-10 | -2.306e+01 | 0.14% | 0.03% | 84.5bp (77.1bp) | NR1I3/MA1534.1/Jaspar(0.688) More Information \| Similar Motifs Found | motif file (matrix) |
| 33 \* | A C T G A C T G A C G T A G T C C G T A A C T G A C T G A C G T A C T G C G T A | 1e-9 | -2.155e+01 | 0.07% | 0.01% | 89.9bp (45.3bp) | SREBF1(var.2)/MA0829.2/Jaspar(0.813) More Information \| Similar Motifs Found | motif file (matrix) |
| 34 \* | A G T C A C G T A C G T A T G C T G C A A G T C A C T G A C G T | 1e-8 | -1.993e+01 | 1.71% | 1.21% | 82.9bp (88.3bp) | HIF2a(bHLH)/785\_O-HIF2a-ChIP-Seq(GSE34871)/Homer(0.732) More Information \| Similar Motifs Found | motif file (matrix) |
| 35 \* | A C T G A C G T C G T A A G T C A C T G A C T G A T G C A C T G | 1e-6 | -1.594e+01 | 1.10% | 0.75% | 82.1bp (90.6bp) | PB0113.1\_E2F3\_2/Jaspar(0.649) More Information \| Similar Motifs Found | motif file (matrix) |
| 36 \* | A C G T A C T G A G T C A C T G A C G T C G T A A T G C C G A T C T A G A G T C T A G C A C T G A C G T | 1e-6 | -1.539e+01 | 0.04% | 0.00% | 82.9bp (45.0bp) | CEBPG/MA0838.1/Jaspar(0.557) More Information \| Similar Motifs Found | motif file (matrix) |
| 37 \* | C G T A A G T C A T C G A C T G A G T C C T G A A G T C A C T G C T A G A G T C C G T A A G T C A C T G | 1e-5 | -1.284e+01 | 0.08% | 0.02% | 63.4bp (91.9bp) | PB0091.1\_Zbtb3\_1/Jaspar(0.622) More Information \| Similar Motifs Found | motif file (matrix) |
| 38 \* | G C A T T A C G A C G T A T G C A T C G A G T C C T A G C T G A | 1e-5 | -1.249e+01 | 1.73% | 1.33% | 87.9bp (87.5bp) | ZBED1/MA0749.1/Jaspar(0.824) More Information \| Similar Motifs Found | motif file (matrix) |
